# Estimation of Immune Cell Densities in Immune Cell Conglomerates: An Approach for High-Throughput Quantification

**DOI:** 10.1371/journal.pone.0007847

**Published:** 2009-11-16

**Authors:** Niels Halama, Inka Zoernig, Anna Spille, Kathi Westphal, Peter Schirmacher, Dirk Jaeger, Niels Grabe

**Affiliations:** 1 Medical Oncology, National Center for Tumor Diseases, University of Heidelberg, Heidelberg, Germany; 2 Hamamatsu Tissue Imaging and Analysis (TIGA) Center, Institute for Medical Biometry and Informatics, University of Heidelberg, Heidelberg, Germany; 3 Institute of Pathology, University of Heidelberg, Heidelberg, Germany; Health Canada, Canada

## Abstract

**Background:**

Determining the correct number of positive immune cells in immunohistological sections of colorectal cancer and other tumor entities is emerging as an important clinical predictor and therapy selector for an individual patient. This task is usually obstructed by cell conglomerates of various sizes. We here show that at least in colorectal cancer the inclusion of immune cell conglomerates is indispensable for estimating reliable patient cell counts. Integrating virtual microscopy and image processing principally allows the high-throughput evaluation of complete tissue slides.

**Methodology/Principal findings:**

For such large-scale systems we demonstrate a robust quantitative image processing algorithm for the reproducible quantification of cell conglomerates on CD3 positive T cells in colorectal cancer. While isolated cells (28 to 80 µm^2^) are counted directly, the number of cells contained in a conglomerate is estimated by dividing the area of the conglomerate in thin tissues sections (≤6 µm) by the median area covered by an isolated T cell which we determined as 58 µm^2^. We applied our algorithm to large numbers of CD3 positive T cell conglomerates and compared the results to cell counts obtained manually by two independent observers. While especially for high cell counts, the manual counting showed a deviation of up to 400 cells/mm^2^ (41% variation), algorithm-determined T cell numbers generally lay in between the manually observed cell numbers but with perfect reproducibility.

**Conclusion:**

In summary, we recommend our approach as an objective and robust strategy for quantifying immune cell densities in immunohistological sections which can be directly implemented into automated full slide image processing systems.

## Introduction

In situ immunohistochemical staining of tumor-infiltrating immune cells against the immune cell surface molecules CD3, CD8, CD45RO and Granzyme B in large cohorts of human colorectal cancers [1]–[3] supports the hypothesis that the adaptive immune response influences the behavior of human tumors. It is important to note that the observed immune cell densities were better predictors of prognosis than the classical TNM classification [4]–[6], initiating a debate on the feasibility of individualized prognosis prediction based on immune cell densities. Early data also indicates a relation of immune cell density to chemotherapy efficacy [7], making detailed quantification of immunologic tumor infiltrating cells even more attractive for clinical decisions. Tumor-infiltrating immune cells therefore represent a valuable prognostic tool in the treatment of colorectal cancer, a high density of immune cells being associated with good outcome independently of other established prognostic markers. In other tumor entities the prognostic value of these immune cells could also be demonstrated [8]–[12]. Immunohistochemical quantitative analysis of immune cell surface markers can therefore be regarded as an important prognostic and predictive tool, requiring a high standard of precision and reproducibility for individualized patient care.

Virtual microscopy (VM) represents an important technological advancement in histology as it allows for the first time the automated high throughput microscopy of complete microscopic slides. By its ability to automatically microscope a full glass slides, VM can deliver unprecedented spatial expression data, visualizing spatial heterogeneity of histological parameters on the level of the individual patient. The capabilities of the new VM technology facilitate the solution of some long standing problems in diagnostic histology.

**Figure 1:**
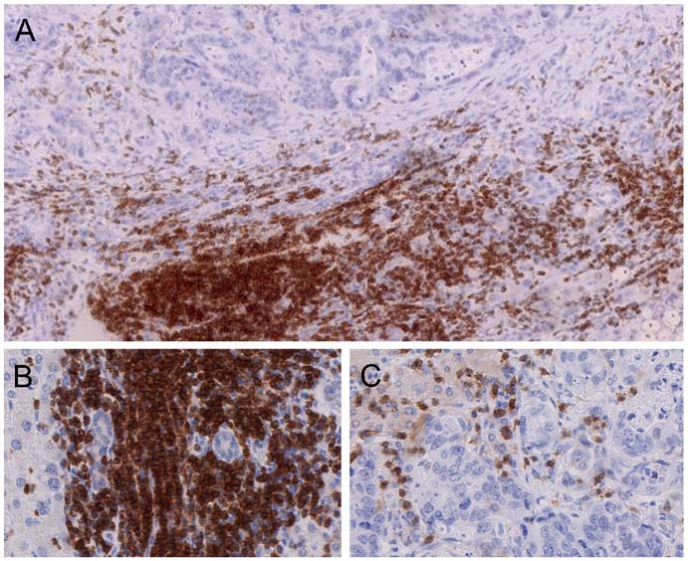
Conglomerates and single cells. Liver metastasis of colorectal cancer with strong T cell infiltrate (CD3 staining: dark red with hematoxylin counterstaining, A: overview, digital magnification 10×, B: conglomerate (magnification 40×), C: single cells, (magnification 40×).

Here we study the contribution of VM to the quantitative analysis of immune cell densities which is regularly obstructed by the presence of immune cell conglomerates. Traditionally, manual cell counts by observers are regarded as the gold standard in histopathological quantification. But for independent observers it is frequently rather difficult to reproduce quantities of cell densities roughly estimated by others [13], [14]. This is especially the case with the difficult estimation of the number of cells contained in complex cell conglomerates (see figure 1), leading to incomplete evaluations of slides [15]. Instead, combining full slide scanning by VM with automatic whole slide image processing provides the basis for developing an algorithmic solution for estimating these cell conglomerates. On the one hand such an algorithm allows the automatic evaluation of large batches of immunohistological slides in an automatic high-throughput manner. On the other hand, it also ensures the comprehensive, objective, reproducible and quantitative evaluation of the respective slides. In this article we present such an algorithmic approach which yields robust and reliable estimations of cell densities in even in large cell conglomerates.

## Materials and Methods

### Tissue Selection

The presented analysis comprises 20 samples from colorectal cancer primary tumors, 12 liver metastases, 10 normal colon mucosa and 10 liver samples. Approval from the medical ethics committee at the University of Heidelberg was obtained and written informed consent was obtained from all patients.

### Immunohistochemical Staining

Tissue specimens were immunohistochemically analyzed for their overall infiltration with T cells (CD3-positive cells). Tissue sections (2 µm) were prepared from formalin-fixed, paraffin-embedded material. After deparaffinization and rehydration, the slides were boiled in 10 mM citrate buffer (pH 6) for 15 minutes to retrieve the antigens. The endogenous peroxidase activity was blocked by incubation with 0.6% H_2_O_2_ in methanol for 20 minutes. The sections were blocked with 10% normal horse serum (Vectastain® Elite ABC kit, Vector, USA). Mouse monoclonal antibodies recognizing human CD3 (1∶50 dilution, clone PS1, Acris, Germany). This antibody was applied as primary antibody at room temperature for 2 hours. The slides were incubated with a biotinylated secondary antibody (1∶50 dilution, horse-anti-mouse IgG, Vectastain® Elite ABC kit, Vector, USA) for 30 minutes at room temperature and AB reagent was applied according to the manufacturer's instructions (Vectastain® Elite ABC kit, Vector, USA). The antigen detection was performed by a color reaction with 3,3-di-amino-benzidine (DAB+ chromogen, DakoCytomation, USA). The sections were counterstained with hematoxylin (AppliChem, Germany) and mounted with Aquatex (Merck, Germany).

### Evaluation of Immune Cell Densities

From twenty sections across primary colorectal cancer, ten sections of normal colon mucosa, ten sections of normal liver and twelve sections of liver metastases a total of 100 samples of single CD3 positive T cells were measured manually for their area (using the Hamamatsu NDP viewer software).

All slides were scanned using the NDP Nanozoomer HT from Hamamatsu Photonics. The NDP Nanozoomer produces virtual images of full tissue scans which have been analyzed visually as well as by automatic image processing algorithms. The full tissue sections allow large scale histological evaluations with high precision across the complete section. Thus, ambiguities due to varying cell densities across the tissue can be avoided. We used a resolution of 0.46 µm/pixel (40×).

Visual analysis of the slides was done using the Hamamatsu NDP viewer. At the invasive margin of colorectal cancer metastases or primary tumors, regions with visually high immune cell densities and conglomerates (each field with 1 mm^2^ size) were selected randomly. Manual evaluation of stained immune cells was performed (in duplicates) by two independent observers. Variations in the identified cell quantities between observers were noted (see table 1). The results for each observer were expressed as the number of positive stained cells/mm^2^. Despite the fact that many different image processing systems could be used, we here implemented the procedure for assessing immune cell densities in cell conglomerates using the Visiomorph software (VisioMorph, Visiopharm, Denmark).

**Table 1:** Cell counts of T cells (CD3+) in thirty different fields of 1 mm^2^ size.

**Field #**	**1**	**2**	**3**	**4**	**5**	**6**	**7**	**8**	**9**	**10**
Manual1	157	184	223	336	384	387	375	648	672	493
Manual2	92	171	207	238	272	422	409	617	476	457
Automated	80	172	208	252	288	366	406	486	504	524
Abs. Diff. Man.	65	13	16	98	112	35	34	31	196	36
% Diff. Man.	41%	7%	7%	29%	29%	9%	9%	5%	29%	7%
**Field #**	**11**	**12**	**13**	**14**	**15**	**16**	**17**	**18**	**19**	**20**
Manual1	546	600	692	751	700	729	854	872	924	1080
Manual2	502	571	642	697	738	895	605	978	748	855
Automated	530	574	654	710	764	789	798	847	852	860
Abs. Diff. Man.	44	29	50	54	38	166	249	106	176	225
% Diff. Man.	8%	5%	7%	7%	5%	23%	29%	12%	19%	21%
**Field #**	**21**	**22**	**23**	**24**	**25**	**26**	**27**	**28**	**29**	**30**
Manual1	814	906	1214	991	1051	1172	1383	1472	1255	1981
Manual2	858	1221	933	1381	1484	1078	1190	1106	1514	2089
Automated	888	963	985	1073	1117	1121	1213	1313	1334	2161
Abs. Diff. Man.	44	315	281	390	433	94	193	366	259	108
% Diff. Man.	5%	35%	23%	39%	41%	8%	14%	25%	21%	5%

Fields with maximum differences between manual cell counts are highlighted.

Abs. Diff. Man. = Absolute Difference between Manual1 and Manual2, % Diff. Man. = Percentage Difference between Manual1 and Manual2.

### Statistical Evaluation

Statistical analyses were performed with SPSS 16.0 software (SPSS, Chicago, IL). For the two-sample comparison of the distribution of continuous variables, exact Mann-Whitney U-tests were used. When comparing different tissues, the exact Kruskal-Wallis test was used. Results with two-tailed P values <.05 were judged to be statistically significant.

## Results

### Measurement of Cell Area Sizes in Sections with Isolated CD3 Positive T Cells

From twenty sections across primary colorectal cancer, ten sections of normal colon mucosa, ten sections of normal liver and twelve sections of liver metastases a total of 100 samples of single CD3 positive T cells were measured manually for their area (see methods). To avoid specific tissue biases, a quarter of the measurements was done in liver tissue, a quarter in metastatic tissue, one quarter in colorectal primary tumors and one quarter in normal mucosa. No significant differences in area sizes were seen between the four tissues (Kruskal-Wallis test, p = 0.581). The resulting average area of an isolated T cell comprised 51 µm^2^ (range being 28–80 µm^2^; standard deviation 10.65 µm^2^). The details on all measured cell areas in different tissues can be found in table 2.

**Table 2:** Cell area sizes of T cells (CD3+) measured in histological sections of different tissues with 2 µm thickness.

Liver	Liver metastases	Primary CRC	Mucosa
57,2	52,6	39,9	53,2
62	47,8	50,7	61
62,6	64,7	37,8	49,3
29	47,7	46,4	66,1
33,4	53,6	32,4	51,6
48,8	53	40,8	63,6
43,4	59	62,6	50
54,8	41,3	80,1	49,6
58,8	49,7	53,8	59,8
41,1	62,2	47,6	69
46,8	43,4	55,3	70,5
42,6	46,6	75,9	47,2
60,2	46,3	58,2	46,6
56,2	63,2	48,1	37,8
42,7	37,1	67,3	64
64,2	59	57,6	60,1
61,3	48	45,5	36,7
50,5	33,9	59,3	39,3
40,7	37,3	51,1	38
51,2	46,8	46,8	56,2
63,7	46,9	50,5	52,9
46,9	44,8	61,3	42,4
39,8	46,7	76	27,5
42,6	62,4	55,6	38,3
31,5	50,1	50	59,3

### Comparison of the Manual Cell Counts for Two Observers on Cell Conglomerates

**Figure 2:**
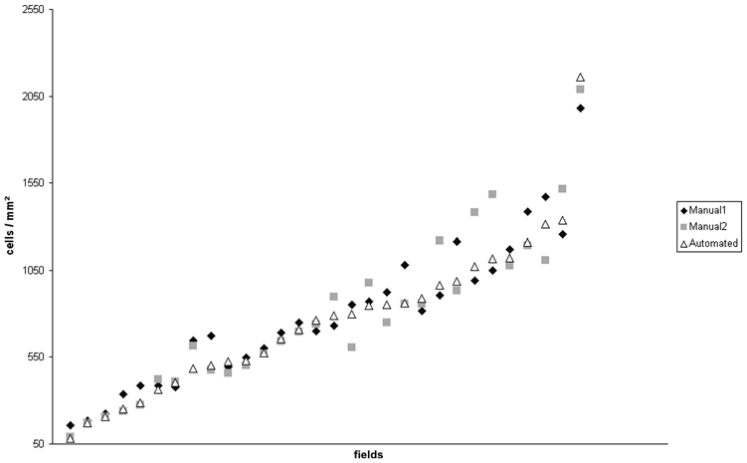
Cell counts for 30 different fields with one ore more conglomerates, each evaluated by two observers (“Manual1” and “Manual2”) and the here presented algorithm (“Automated”). Note the up to 41% variation between the observers at high cell counts (1000-1.500). For quantitative data comparison see table 1.

**Figure 3:**
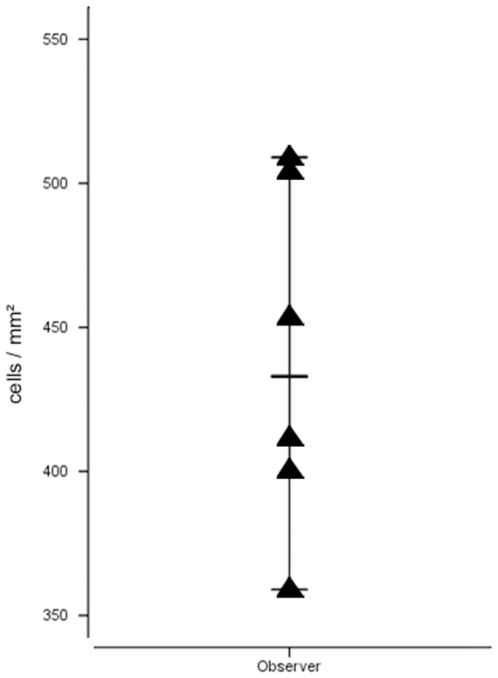
Repeated quantification (six times) of a large conglomerate by one observer. Triangles show single values for each repetition and thin vertical lines indicate range, thick horizontal line indicates average value.

**Figure 4:**
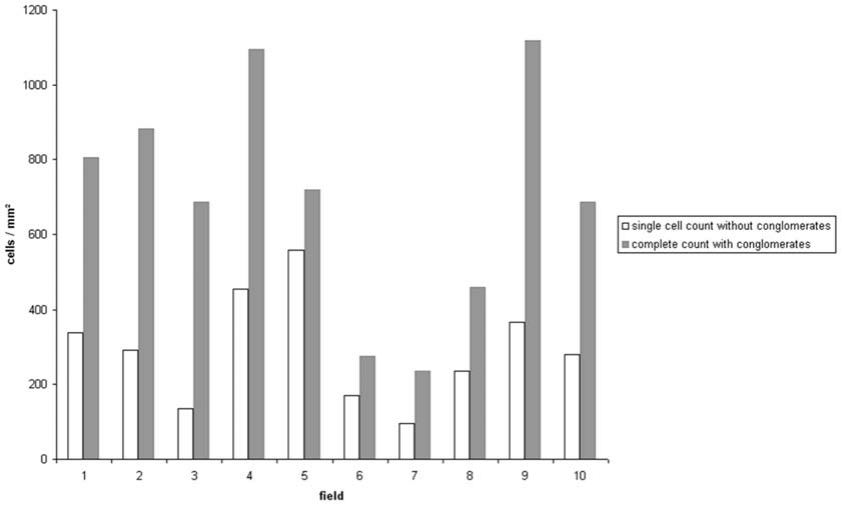
Impact of cell counts from conglomerates on total cell counts in 10 individual 1 mm^2^ fields from sections of 10 different patients. Omitting conglomerates in the quantification would substantially distort the total cell counts.

Figure 2 depicts the results for the individual observers and for the automated cell counting algorithm. Table 1 shows the according quantitative values measured for the two observers and the described automated algorithm as well as the observed differences. With increasing numbers of stained cells the divergence between the two observers with respect to the resulting cell densities was remarkable (partly exceeding 400 cells/mm^2^ difference). We calculated the variability as ranging from 5 to 41% depending on the area size of the conglomerate. As can be seen in figure 1, the estimation of cell densities in conglomerates by visual inspection is extremely difficult (see figure 1B) which explains the partly large differences in the manual evaluations. Especially in large conglomerates the estimation is not only time consuming but also extremely biased by the individual observer's ability to estimate area sizes. The observers were allowed to repeat their calculations but this did not diminish the variability. A representative example is shown in figure 3, where the observer repeated the cell count for a single conglomerate six times. As therapy selection is increasingly done depending on exact thresholds, this clearly emphasizes the above mentioned fundamental need for a reliable method capable of objectively estimating cell densities in conglomerates. To estimate the importance of also incorporating conglomerates (besides single cell counts) into quantitative immunhistological cell counts compared to only considering the single cells we analyzed 10 fields of 10 different patients for single cells and for conglomerates (figure 4). The data show massive differences between the complete analysis and single cell counts only, rendering the inclusion of conglomerates in quantitative slide evaluations mandatory.

### Details of the Automated Algorithm

**Figure 5:**
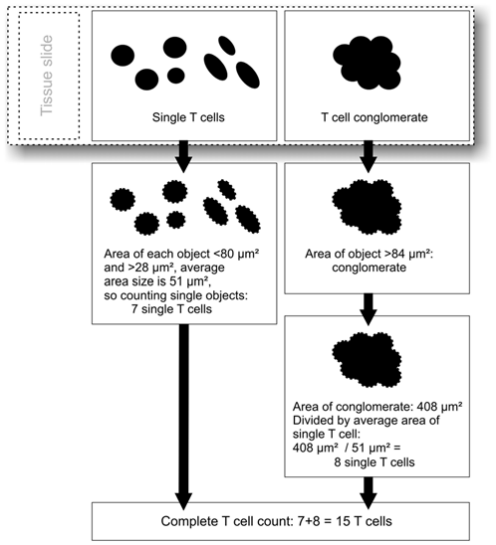
Exemplary workflow for the described algorithm. Stained immune cells are either counted individually (where possible) or the number of cells is estimated by the conglomerate surface. Both results are added.

The schematic approach is depicted in figure 5. The here described conglomerate and cell quantification procedure is embedded in a complex automated image processing pipeline including segmentation, color deconvolution and analyses of cellular morphology. The detailed implementation can vary between different computational image processing systems (Image J, VIS, Definiens, Alphelys etc.) and the respective implementation details are beyond the scope of this paper. Moreover, such algorithms are dependent on the specific tissue the respective target antigen is studied in. Specific explanations regarding image processing are therefore omitted here, because any implementation should somehow lead to the detection of single cells and aggregated cell clusters (“conglomerates”). Instead, here we present a general procedure for dealing with conglomerates in quantitative tissue analysis, independent of individual systems.

In dependence on the immunohistological detection system, every CD3 positive T cell is specified by a characteristic color and shape (“roundness”). These two parameters were used to identify CD3 positive cells in the tissue (“target objects”) either in isolated form or in form of conglomerates. Based on the target objects' area size it was classified into “single T cells” or “T cell conglomerates”. If the area size exceeded triple the observed lower range of area (28 µm^2^ * 3 = 84 µm^2^) the target object was classified as “T cell conglomerates”, smaller objects were regarded as “single T cells” if within the normal range of T cell sizes. “Single T cell” objects were counted per field (1 mm^2^ each). For a “T cell conglomerate” its area was divided by the average area of a single T cell (51 µm^2^) to obtain an estimate of the underlying cell density yielding an estimated T cell number. These T cell numbers were added to the number of “single T cell” objects to obtain a final estimation of cell densities for the given field.

### Comparison of the Results from the Automated Algorithm with the Manually Observed Numbers

Using 10× digital magnification, all manually counted fields (1 mm^2^ each) were re-analyzed using an automated algorithm based on the following approach. The manually obtained cell densities varied greatly across the two observers and accounted for a deviation of the estimated cell numbers of approximately 10%. The results are in line with other reports on inter-observer variability in histology stating even higher deviations between two observers [13]. This marked high inter-observer deviation is avoided with the automated system. Figure 2 shows the observed cell numbers for two independent observes in context of the performed corresponding automated analysis. Each field is 1 mm^2^ and contains one or more conglomerates and scattered single CD3 positive T cells.

## Discussion

**Figure 6:**
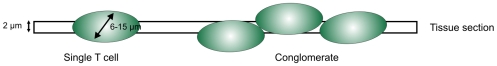
Rationale behind the presented approach. The figure shows the relation of the size of T cells to the height of the used tissue sections (2 µm). The sections are so thin, that there is only minimal overlap between the individual cells of a conglomerate. This allows for calculating the number of cells in a conglomerate by its total area.

In diagnostic pathology the accurate determination of cell counts is of substantial importance as histological cut-offs are increasingly used as a basis for determining a patient's individual therapy strategy. Therefore, quantitative and objective methods are of primary concern in diagnostics and clinical decisions [16], especially in light of the accelerating automation of diagnostic routines. More and more image processing algorithms are used for an automatic pre-evaluation of tissue slides as a support for the diagnosing pathologist [17]. In immunological evaluations, cell counting is generally limited by the underlying quality and structure of the studied tissue sections. For example, overlapping cells are of concern when assessing tissue sections of greater thickness. The sections used here had a thickness of 2 µm, thus minimizing the possible overlap between immune cells. T cells have a diameter of 6 to 15 µm. Therefore, the here described procedure is untroubled also by densely packed cell conglomerates (see figure 6).

We here dealt with the problem of incorporating complex cell conglomerates into histological cell count cut-off studies. The presented data shows an enormous impact of conglomerates on calculated cell densities for a given field (see figure 4). Simply ignoring conglomerates as it is done in some recent studies [15] is not acceptable because this leads to severe bias, which is especially devastating in clinically relevant settings where accurate evaluation with regard to cut-offs may be crucial to patient treatment or survival. In our view accurate counting also in conglomerates is indispensible for determining quantitative immunological patient responses. Image processing approaches including complex mathematical operations dissecting the conglomerates by finding local minimal and maximal staining intensities in an image (“watershed algorithms”) could be considered, but generally are prone to severe errors due to unavoidable staining variability. Our approach here uses the reliable statistical and biological basis of immune cell size to calculate cell densities in conglomerates. It is applicable with widely used available software image analysis systems (ImageJ, VisioMorph, Definiens, etc.) and can implemented straight forward. The rather straight-forward mathematical image processing operations avoid complex computational operations and parameter settings. In this respect, our approach is very attractive especially for high-throughput quantifications of immunohistological evaluations. In summary we have shown that (1) quantification of immune cell conglomerates is indispensable when quantitatively evaluating immunohistological slides for immune cell markers and (2) an intuitive, high-throughput capable procedure for the objective and robust quantification of immune cell conglomerates.
